# Metabolic Syndrome Prevalence in Students Attending West Virginia University

**DOI:** 10.3390/jcm7120487

**Published:** 2018-11-27

**Authors:** Melissa D. Olfert, Amanda Dent, Rachel A. Wattick

**Affiliations:** Division of Animal and Nutritional Sciences, Davis College of Agriculture, Natural Resources and Design, West Virginia University, 1194 Evansdale Dr., Office G27, Morgantown, WV 26506, USA; amandamariedent@gmail.com (A.D.); rawattick@mix.wvu.edu (R.A.W.)

**Keywords:** metabolic syndrome, Appalachia, college students, metabolic syndrome

## Abstract

Metabolic Syndrome (MetS) contributes to the development of cardiovascular disease (CVD) and type II diabetes mellitus (T2DM). Few studies have investigated the MetS risk of young adults (18–24 years old). This study aims to determine the prevalence of MetS in Appalachian and non-Appalachian students attending West Virginia University. The prevalence of MetS in this population was 15%. There was no difference in MetS prevalence between male students and female students (18.8% males and 11.1% females, *p*-value = 0.30), or between Appalachian students and non-Appalachian students (17.7% Appalachian and 10.0% non-Appalachian, *p*-value = 0.33). Identification of MetS early in life is needed in order to reduce the onset of chronic disease. Therefore, implementing a screening process to identify at-risk young adults will help tailor more effective behavioral interventions.

## 1. Introduction

Metabolic Syndrome (MetS) contributes to the development of coronary heart disease (CHD) [1,2,3], cardiovascular disease (CVD) [3], and type II diabetes mellitus (T2DM) [1,2,3]. MetS consists of five, interrelated risk factors [4,5]. Individuals presenting three or more of the following risk factors are diagnosed with MetS: Fasting blood glucose (FBG) ≥100 mg/dL, waist circumference (WC) >102 cm for men and >88 cm for women, triglycerides (TG) ≥150 mg/dL, high density lipoprotein cholesterol (HDL) <40 mg/dL for men and <50 mg/dL for women, and blood pressure (BP) ≥130/85 mmHg [6].

Few studies have investigated the MetS risk of young adults [1,7]. However, CHD is the second leading cause of death in this age group [1], suggesting the need to detect for early warning signs of this disease. Thus, screening for MetS in this population can be beneficial in identifying those at higher risk from CHD development.

The prevalence of obesity has reached epidemic proportions within the Appalachian region [8,9], which extends from Northeastern Mississippi to Southern New York [10], with its epicenter lying entirely within the state of West Virginia (WV) [11]. The Appalachian region is characterized by unhealthy eating behaviors and inactivity, which leads to the increased incidence of chronic disease among this population [10]. Ely et al. recently reported the increased risk of chronic disease, related to excessive weight gain and poor health behaviors, is not realistically perceived by Appalachian residents [12]. Inadequate transportation [8], poverty [13,14], lack of access to medical care [8,13] and lack of insurance are additional factors directly impacting the health disparities of individuals in this region [13]. In 2009, Appalachians were 40% more likely to have diabetes than non-Appalachians [8]. In 2011, the prevalence of diagnosed diabetes was 9.8% within the Appalachian region and 7.8% in the rest of the nation [15].

In Ohio and WV, 25.6% of normal-weight adults display two or more of the following cardio-metabolic abnormalities: High blood pressure, elevated TG, decreased HDL, elevated FBG, and insulin resistance (IR) [9]. An estimated 50% of adults and adolescents residing in WV are obese by Body Mass Index (BMI) classification: Normal weight (18.5–24.9 kg/m^2^), overweight (25.0–29.9 kg/m^2^), and obese (≥30 kg/m^2^) [8]. WV has reported the highest age-adjusted incidence of diabetes as well as the highest diabetes-related deaths in the nation [8].

As many as 30% to 35% of college students are reported to be overweight or obese [7,16,17] and obesity rates have increased most rapidly among individuals 18–29 years old with some college education [7,16,17,18]. First year college students gain weight up to 11 times faster than adults [1]. This increase in weight increases the likelihood of developing MetS risk criteria [1,7]. Increased weight and obesity, particularly abdominal obesity, is directly related to the development of MetS and cardiovascular risk [5,19]. Screening for abnormal lipid values is recommended starting at 20 years old [1]. Therefore, colleges and universities serve as important settings for the surveillance, prevention, and intervention of hyperlipidemia, MetS, and CHD [20]. Targeting young adults that choose to attend post-secondary institutions could aid in the prevention of CVD, CHD, and T2DM since many young people develop a clearer sense of self and establish life-long behavior patterns during college [21].

The few studies of MetS prevalence in college students found that 27% [17], 28% [1], and 30% [16] of students in the Northeast [1] and Midwest [16,17] regions exhibit at least one component of MetS. In the same studies, 5.7% [16], 7.4% [1], and 14.3% [17] of college students had two components of MetS. In the Southern US, 43% of students presented with at least one component of MetS [7]. When the sample was limited to students who were overweight or obese, the rate of at least one component of MetS rose to 82% [7]. Fernandes et al. found that 3.7% of college students presented with three or more MetS risk components [1]. The latest study, conducted by Morrell et al., reported 77.2% of males and 53.8% of females exhibited at least one component of MetS [22]. MetS was present in 9.9% of males and 3.0% females [22]. Not only do overweight [1,17,22] and obese [1,22] students present with MetS [1] and more MetS risk factors [17,22] than normal weight students, but obese students exhibit significantly more MetS criteria than overweight students [22]. Overall, male students were more likely to exhibit MetS risk criteria than female students [16] and the most prevalent MetS risk component was low HDL [16,17,22], then FBG, and TG in decreasing order [16,17].

Due to the overall poorer health and higher risk factors in the Appalachian region discussed previously, young adults in these regions are an important, yet overlooked, demographic to study their MetS risk. This study aims to determine the prevalence of MetS in Appalachian and non-Appalachian students attending West Virginia University.

## 2. Method

The measures for this cross-sectional study were collected in January and February 2011. Subjects were recruited from West Virginia University. The Institutional Review Board (IRB) at West Virginia University approved the study protocol in advance of commencement.

### 2.1. Subjects

Subjects (*n* = 93) were recruited via posted advertisements, in-class announcements, and recruitment E-mails sent by instructors and campus administrators. Students were eligible if they were 18–24 years old; had a BMI >18.5 kg/m^2^; were a first, second, or third year undergraduate; had regular access to the Internet; were free from life-threatening illnesses or other health conditions; were not pregnant; had no diet- and/or activity-related medical restrictions that prevented accurate physical assessments; they were not currently enrolled in a nutrition course; and they were not majoring in nutrition, exercise science, or health-promotion.

### 2.2. Measures

Demographics (e.g., ethnicity), health-related practices (e.g., cigarette and medication use), and Appalachian identity (from self-reported home address) were collected via questionnaire. All clinical, anthropometric, and biochemical measurements were performed by trained staff. Measurements were collected in the fasted state (>8 h) after participants had changed into light clothing and voided. All female participants were screened for pregnancy.

Systolic and diastolic pressures were measured in triplicate (with 2 min rest intervals) in the left arm midpoint between the shoulder and elbow via an automated cuff (HEM-907XL, Omron; Lake Forest, IL, USA) after subjects were rested in a seated position for five minutes. Height was measured by a wall-mounted, digital stadiometer (Heightronic 235, Quick Medical; Issaquah, WA, USA) with subjects looking straight ahead and maintaining four points of contact (heels, buttocks, shoulder blades, and back of the head) with the wall. Weight was measured by a digital scale (300A, Tanita; Arlington Heights, IL, USA) which also calculated body mass index (BMI) from height input. Waist circumference (WC) was measured in duplicate at the top of the iliac crest to the nearest 0.1 cm using a non-stretchable tape measure with tensometer (Gulick, Creative Health Products; Plymouth, MI, USA). Neck circumference (NC) was measured in duplicate at the point below the larynx to the nearest 0.1 cm with the tape measure used for waist circumference.

Biochemical measures were obtained via finger-stick using a desktop LDX Cholestech Analyzer (LDX, Cholestech; San Diego, CA, USA) following manufacturer’s instructions. Analyses for high-density lipoprotein cholesterol (HDL), triglycerides (TG), fasting blood glucose (FBG), C-Reactive Protein (CRP), and low-density lipoprotein cholesterol (LDL) were conducted via direct enzymatic methods by the analyzer. The Cholestech LDX analyzer has been validated for precision and accuracy against traditional laboratory techniques and offers logistical convenience for large-scale health assessments [23]. Hemoglobin A1C (HbA1C) was measured using DCA Vantage. Hemoglobin (Hgb) and hematocrit (Hct) weer measured using HemoPoint A2 (Boerne, TX, USA).

### 2.3. Statistical Analysis

BMI was categorized as normal weight (18.5–24.9 kg/m^2^), overweight (25.0–29.9 kg/m^2^), and obese (≥30.0 kg/m^2^). MetS and its five individual components were defined by the National Cholesterol Education Program Adult Treatment Panel III: Fasting blood glucose (FBG) ≥100 mg/dL, waist circumference (WC) >102 cm for men and >88 cm for women, triglycerides (TG) ≥150 mg/dL, high density lipoprotein cholesterol (HDL) <40 mg/dL for men and <50 mg/dL for women, and blood pressure (BP) ≥130/85 mmHg [6]. The presence of ≥3/5 components was categorized as MetS.

Student *t*-tests were performed to identify differences in anthropometric and biochemical measures between males and females and Appalachians and non-Appalachians. Chi-square tests were used to identify associations between prevalence rates of individual components and MetS, and the following subject characteristics: Male, female, Appalachian, and non-Appalachian. The Mantel-Haenszel test was used to assess the ordinal trend between males and females as well as Appalachians and non-Appalachians. Statistical significance was defined as a *p*-value < 0.05. Statistical analyses were performed using SAS 9.3 statistical software (SAS Institute Inc., Cary, NC, USA).

## 3. Results

Subjects were 51.6% male and 48.4% female; primarily White (82.8%) followed by Black (10.7%), Latino (3.2%) and Asian (2.2%). Roughly, two thirds of the subjects identified themselves as Appalachian (67.7%) and one third as non-Appalachian (32.3%). The majority (41.9%) of subjects were first year, 30.1% were second year, and 25.8% third year undergraduate students. Table 1 provides more descriptive characteristics.

Table 2 shows that average subjects had normal BMIs with no significant differences between male and female) subjects (*p*-value = 0.29). Waist circumference did not differ between male and female subjects (*p*-value = 0.58) (Table 2). Males weighed significantly more than females (*p*-value < 0.01) (Table 2). Female students presented with significantly higher HDL levels compared to males (*p* < 0.01) (Table 3). Male students had higher systolic BP (*p*-value < 0.001), but not diastolic BP than females (*p*-value = 0.60) (Table 3). No sex differences were observed for TG and FBG (Table 3).

Appalachian and non-Appalachian student comparisons revealed no significant differences in anthropometric measures (height, weight, BMI, and WC) or biochemical measures (HDL, TG and FBG) (Table 4). Appalachian students presented with elevated FBG (37.1%), low HDL (35.5%), at-risk WC (21.0%), elevated TG (21.0%), and elevated BP (19.4%). Non-Appalachian students presented with elevated FBG (36.7%) low HDL (36.7%), elevated BP (23.3%), at-risk WC (16.7%) and elevated TG (13.3%) (Table 5). The prevalence of individual MetS components was not significantly different between Appalachian and non-Appalachian students.

The most prevalent MetS component in the total sample was elevated FBG (37.6%), followed by low HDL (35.5%), elevated BP (20.4%), at-risk WC (19.4%) and elevated TG (18.3%) (Table 5). Male students presented with elevated FBG (47.9%), followed by low HDL (39.6%), elevated BP (31.3%), elevated TG (16.7%), and at-risk WC (8.3%), whereas, female students presented with low HDL (31.1%) and at-risk WC (31.1%), followed by elevated FBG (26.7%), elevated TG (20.0%), and elevated BP (8.9%) (Table 5). Overall, significantly more female students exhibited at-risk WC than males (*p* < 0.01), whereas, significantly more male students displayed elevated BP (*p* < 0.01) and FBG (*p* < 0.05) (Table 5).

Twenty-eight percent of students presented with zero components of MetS; 33.1% with one component; and 23.7% with two components (Table 6). The prevalence of MetS in this population was 15%. Specifically, 10.8% presented with three components, 3.2% with four components, and 1.1% with all five components of MetS (Table 6). There was no significant difference between male and female students in MetS prevalence (*p*-value = 0.30) or between Appalachian students and non-Appalachian students (*p*-value = 0.33) (Table 6). There were no significant associations between the number of MetS components by sex (*p* = 0.28) or by Appalachian identity (*p* = 0.78) (Table 6).

## 4. Discussion

The prevalence of MetS students at West Virginia University (15.1%) is much higher than what has been reported in previous studies (0.6% to 10%) of MetS prevalence in college students [1,7,16,17,22]. Previous studies have reported the prevalence of MetS in young adults to be 0.6% [17] and 1.3% [16] in the Midwest region, 3.7% [1] in the Northeast region and up to 10% in the Southeast region. Additionally, in the Northeast 7% [1]; and in the Midwest 14% [17] and 6% [16] of students had two MetS risk factors, while 24% had two MetS risk factors in the Appalachian region. Showing that Appalachia has a markedly higher incidence of MetS [1,7,16,17,22] and MetS risk factors [1,16,17] than other regions of the US.

In a direct comparison of West Virginia University students to students from two Northeast universities (NEU1 and NEU2), the prevalence of MetS was significantly greater at WVU than both NEU1 (*p*-value < 0.05) and NEU2 (*p*-value < 0.05) [24]. The surprising lack of a significant difference between Appalachian and non-Appalachian student identity and the higher prevalence of MetS in WVU students suggests that an unidentified factor may be mediating the relationship with higher incidence of MetS in this population. One possible factor is BMI. Upon examining correlations between BMI and MetS components significant relationships were found. Specifically, BMI was significantly correlated with students presenting with at-risk WC (*R*^2^ = 0.86, *p* < 0.05), low HDL (*R*^2^ = −0.43, *p* < 0.05), elevated BP (*R*^2^ = 0.29, *p* < 0.05) and increased TG (*R*^2^ = 0.22, *p* < 0.05). No correlation was found between BMI and impaired FBG measures.

There are several limitations to this study. First, this was a small sample size of students. While this area of study is important, having a larger sample size would have increased the impact of these results. Overall, there was a generally low prevalence of MetS in this sample. Future work would include a larger sample size to have more impactful results.

These data identify the need for university administrators throughout the nation to implement health initiatives to measure BMI and screen for MetS components in students to prevent the development of chronic disease. Data from such screenings for college students will provide researchers, public health officials, and administrators with information to use to design, tailor, and implement effective interventions to prevent chronic disease progression across various university settings. 

## Figures and Tables

**Table 1:** Sample descriptive characteristics.

	Total (*n* = 93)	Percentage (%)
Sex		
Female	45	48.4
Male	48	51.6
Ethnicity		
Caucasian	77	82.8
African American	10	10.7
Hispanic/Latino	3	3.2
Asian	2	2.2
Not reported	1	1.1
Permanent address		
Appalachian	63	67.7
Non-Appalachian	30	32.3
School year		
First	39	41.9
Second	28	30.1
Third	24	25.8
Not reported	2	2.2

**Table 2:** Baseline anthropometric measures by sex.

	All (*n* = 93)		Female (*n* = 45)		Male (*n* = 48)		Sex Differences
Measures	Mean	SD	Mean	SD	Mean	SD	*p*-Value
Height (cm)	171.87	8.94	165.01	5.20	178.30	6.63	<0.0001 *
Weight (kg)	73.04	15.58	68.74	16.53	77.08	13.61	0.0092 *
BMI (kg/m^2^)	24.70	4.81	25.25	5.82	24.19	3.61	0.2930
BF (%)	22.84	10.64	30.22	8.89	15.91	6.79	<0.0001 *
WC (cm)	84.13	15.73	85.07	19.77	83.25	10.80	0.5796
NC (cm)	35.68	3.70	33.04	3.12	38.17	2.17	<0.0001 *

SD: Standard Deviation; BMI: Body Mass Index; BF: Body Fat; WC: Waist Circumference; NC: Neck Circumference; * indicates significance.

**Table 3:** Baseline biochemical measures by sex.

	All (*n* = 93)		Female (*n* = 45)		Male (*n* = 48)		Sex Differences
Characteristic	Mean	SD	Mean	SD	Mean	SD	*p*-Value
SBP (mmHg)	117.74	13.23	111.27	9.68	123.80	13.30	<0.0001 *
DBP (mmHg)	69.41	8.97	69.91	9.21	68.94	8.82	0.6038
LDL-C (mg/dL)	97.73	29.23	100.18	27.00	95.14	31.60	0.4618
HDL-C (mg/dL)	53.06	18.79	58.84	18.31	47.65	17.76	0.0035 *
TC (mg/dL)	165.72	36.54	176.82	38.06	155.30	32.08	0.004 *
TG (mg/dL)	99.51	57.69	107.38	61.86	92.13	53.06	0.2043
FBG (mg/dL)	98.84	13.87	97.38	15.97	100.21	11.57	0.3280
HBA1c (%)	5.28	0.40	5.31	0.50	5.25	0.29	0.4869

SD: Standard Deviation; SBP: Systolic Blood Pressure; DBP: Diastolic Blood Pressure; LDL-C: Low Density Lipoprotein Cholesterol; HDL-C: High Density Lipoprotein Cholesterol; TC: Total Cholesterol; TG: Triglycerides; FBG: Fasting Blood Glucose; HBA1c: Hemoglobin A1C; * Indicates Significance.

**Table 4:** Biochemical and anthropometric measures by region.

	All (*n* = 93)		Appalachian (*n* = 62)		Non-Appalachian (*n* = 30)		Regional Differences
Characteristic	Mean	SD	Mean	SD	Mean	SD	*p*-value
Height (cm)	171.87	8.94	170.94	9.00	174.22	8.33	0.097
Weight (kg)	73.04	15.58	73.19	16.78	73.05	13.3	0.968
BMI (kg/m^2^)	24.70	4.81	25.04	5.40	23.99	3.34	0.332
BF (%)	22.84	10.64	23.37	11.27	21.45	9.30	0.420
WC (cm)	84.13	15.37	83.28	13.56	82.67	9.38	0.825
NC (cm)	35.68	3.70	35.51	3.79	36.12	3.58	0.466
SBP (mmHg)	117.74	13.23	117.63	12.92	118.07	14.27	0.833
DBP (mmHg)	69.41	8.97	70.18	8.60	67.93	9.80	0.265
LDL-C (mg/dL	97.93	29.23	101.21	32.39	87.81	16.50	0.076
HDL-C (mg/dL)	53.06	18.79	53.34	19.55	52.50	17.75	0.843
TC (mg/dL)	165.72	36.64	171.39	38.58	152.77	28.43	0.021 *
TG (mg/dL)	99.51	57.69	104.95	60.17	87.00	51.64	0.164
FBG (mg/dL)	98.84	13.87	99.02	15.43	98.30	10.40	0.819
HBA1c (%)	5.28	0.40	5.32	0.44	5.18	0.29	0.115

SD: Standard Deviation; BMI: Body Mass Index; BF: Body Fat; WC: Waist Circumference; NC: Neck Circumference; SBP: Systolic Blood Pressure; DBP: Diastolic Blood Pressure; LDL-C: Low Density Lipoprotein Cholesterol; HDL-C: High Density Lipoprotein Cholesterol; TC: Total Cholesterol; TG: Triglycerides; FBG: Fasting Blood Glucose; HBA1c: Hemoglobin A1C; * indicates significance.

**Table 5:** Rates of individual metabolic syndrome components.

MetS Component	Number (%) with MetS Component (Total *n*/Percentage %)						
	Overall	Males	Females	*p*-Value	App	Non-App	*p*-Value ^1^
Elevated WC	18 (19.4%)	4 (8.3%)	14 (31.1%)	0.0055 *	13 (21.0%)	5 (16.7%)	0.6259
Elevated BP	19 (20.4%)	15 (31.3%)	4 (8.9%)	0.0075 *	12 (19.4%)	7 (23.3%)	0.6586
Elevated TGs	17 (18.3%)	8 (16.7%)	9 (20.0%)	0.6777	13 (21.0%)	4 (13.3%)	0.3765
Elevated FBG	35 (37.6%)	23 (47.9%)	12 (26.7%)	0.0345 *	23 (37.1%)	11 (36.7%)	0.968
Low HDL-C	33 (35.5%)	19 (39.6%)	14 (31.1%)	0.3935	22 (35.5%)	11 (36.7%)	0.9117

App: Appalachian; WC: Waist Circumference; BP: Blood Pressure; TGs: Triglycerides; FBG: Fasting Blood Glucose; HDL-C: High Density Lipoprotein Cholesterol; ^1^ Chi-Square test; * indicates significance.

**Table 6:** Frequency of metabolic syndrome and number of metabolic syndrome components.

Demographic Variable	*n*	Total *n* (%) with MetS	*p*-Value ^1^	Number of MetS Components (Total *n*/Percentage %)						*p*-Value ^2^
				0	1	2	3	4	5	
Overall	93	14 (15.1%)	-	26 (28.0%)	31 (33.3%)	22 (23.7%)	10 (10.8%)	3 (3.2%)	1 (1.1%)	
Sex			0.3033							0.2770
Males	48	9 (18.8%)		12 (25.0%)	15 (33.3%)	12 (25.0%)	6 (12.5%)	3 (6.3%)	0 (0.0%)	
Females	45	5 (11.1%)		14 (31.1%)	16 (35.6%)	10 (22.2%)	4 (8.9%)	0 (0.0%)	1 (2.2%)	
Region			0.3325							0.7795
Appalachian	62	11 (17.7%)		18 (29.0%)	19 (30.7%)	14 (22.6%)	9 (14.5%)	1 (1.6%)	1 (1.6%)	
Non-Appalachian	30	3 (10.0%)		8 (26.7%)	11 (36.7%)	8 (26.7%)	1 (3.3%)	2 (6.7%)	0 (0.0%)	

MetS: Metabolic Syndrome; ^1^ Chi Square Test; ^2^ Mantel-Haenszel test for trend.

## References

[1] Fernandes J., Lofgren I.E. (2011). Prevalence of metabolic syndrome and individual criteria in college students. J. Am. Coll. Health.

[2] Eckel R.H., Grundy S.M., Zimmet P.Z. (2005). The metabolic syndrome. Lancet.

[3] Grundy S.M., Brewer H.B., Cleeman J.I., Smith S.C., Lenfant C. (2004). Definition of metabolic syndrome. Circulation.

[4] Taylor J.R., Lopez L.M. (2004). Cholesterol: Point-of-care testing. Ann. Pharmacother..

[5] Grundy S.M., Cleeman J.I., Daniels S.R., Donato K.A., Eckel R.H., Franklin B.A., Gordon D.J., Krauss R.M., Savage P.J., Smith S.C. (2005). Diagnosis and management of the metabolic syndrome. Circulation.

[6] Adult Treatment Panel III (2001). Executive summary of the third report of the National Cholesterol Education Program (NCEP) expert panel on detection, evaluation, and treatment of high blood cholesterol in adults (Adult Treatment Panel III). JAMA.

[7] Keown T.L., Smith C.B., Harris M.S. (2009). Metabolic syndrome among college students. J. Nurse Pract..

[8] Pancoska P., Buch S., Cecchetti A., Parmanto B., Vecchio M., Groark S., Paulsen S., Bardwell G., Morton C., Chester A. (2009). Family networks of obesity and type 2 diabetes in rural Appalachia. CTS Clin. Transl. Sci..

[9] Blake K.B., Shankar A., Madhavan S., Ducatman A. (2010). Associations among cardiometabolic risk factor clustering, weight status, and cardiovascular disease in an Appalachian population. J. Clin. Hypertens..

[10] Wu T., Snider J.B., Floyd M.R., Florence J.E., Stoots J.M., Makamey M.I. (2009). Intention for healthy eating among southern Appalachian teens. Am. J. Health Behav..

[11] Griffith B.N., Lovett G.D., Pyle D.N., Miller W.C. (2011). Self-rated health in rural Appalachia: Health perceptions are incongruent with health status and health behaviors. BMC Public Health..

[12] Ely G.E., Miller K., Dignan M. (2011). The disconnect between perceptions of health and measures of health in a rural Appalachian sample: Implications for public health social workers. Soc. Work Health Care.

[13] Williams K.J., Taylor C.A., Wolf K.N., Lawson R.F., Crespo R. (2008). Cultural perceptions of healthy weight in rural Appalachian youth. Rural Remote Health.

[14] Smith S.L., Tessaro I.A. (2005). Cultural perspectives on diabetes in an Appalachian population. Am. J. Health Behav..

[15] Barker L., Gerzoff R., Crespo R., Shrewsberry M. (2011). Age at diagnosis of diabetes in Appalachia. Pop. Health Metr..

[16] Huang T.T.K., Shimel A., Lee R.E., Delancey W., Strother M.L. (2007). Metabolic risks among college students: Prevalence and gender differences. Metab. Syndr. Relat. Disord..

[17] Huang T.T.K., Kempf A.M., Strother M.L., Li C., Lee R.E., Harris K.J., Kaur H. (2004). Overweight and components of the metabolic syndrome in college students. Diabetes Care.

[18] Racette S.B., Deusinger S.S., Strube M.J., Highstein G.R., Deusinger R.H. (2008). Changes in weight and health behaviors from freshman through senior year of college. J. Nutr. Educ. Behav..

[19] Steinberger J., Daniels S.R., Eckel R.H., Hayman L., Lustig R.H., McCrindle B., Mietus-Snyder M.L. (2009). Progress and challenges in metabolic syndrome in children and adolescents. Circulation.

[20] Undén A.-L., Krakau I., Högbom M., Romanus-Egerborg I. (1995). Psychosocial and behavioral factors associated with serum lipids in university students. Soc. Sci. Med..

[21] Rozmus C.L., Evans R., Wysochansky M., Mixon D. (2005). An analysis of health promotion and risk behaviors of freshman college students in a rural southern setting. J. Pediatr. Nurs..

[22] Morrell J.S., Lofgren I.E., Burke J.D., Reilly R.A. (2012). Metabolic syndrome, obesity, and related risk factors among college men and women. J. Am. Coll. Health.

[23] Dale R.A., Jensen L.H., Krantz M.J. (2008). Comparison of two point-of-care lipid analyzers for use in global cardiovascular risk assessments. Ann. Pharmacother..

[24] Morrell J.S., Byrd-Bredbenner C., Quick V., Olfert M., Dent A., Carey G.B. (2014). Metabolic syndrome: Comparison of prevalence in young adults at three land-grant universities. J. Am. Coll. Health.

